# Bacteriological Study of Electronic Devices Used by Healthcare Workers at Ruhengeri Referral Hospital

**DOI:** 10.1155/2020/5872929

**Published:** 2020-06-23

**Authors:** Thierry Habyarimana, Christelle Uwizeye, Emmanuel Munyeshyaka, Cedrick Izere, Joseph Mucumbitsi, Callixte Yadufashije

**Affiliations:** Biomedical Laboratory Sciences Department/Institut d'Enseignement Supérieur de Ruhengeri, PO Box 155 Ruhengeri, Musanze, Rwanda

## Abstract

Electronic devices have become one of the most essential accessories being used in hospitals. Those devices increase the communication and contact making healthcare delivery more efficient and quality service oriented. The study was designed to collect reliable information about the spreading of pathogens through electronic devices especially in sensitive departments. The objectives of this study were to evaluate the bacterial colonization of electronic devices and determine the effectiveness of disinfection with alcohol 70% (*w*/*v*) to reduce the bacterial colonization of electronic devices. It was a cross-sectional study where samples were collected by means of moistened swabs in sterile saline solution from 30 electronic devices used by healthcare workers at Ruhengeri Referral Hospital within four different units: maternity, neonathology, intensive care, and theater room. To evaluate the effects of disinfection using 70% isopropyl alcohol, the second sample collection was carried out after decontamination with 70% isopropyl alcohol. Samples were analyzed in the microbiology lab of INES-Ruhengeri. The result showed that *Staphylococcus aureus* was the most predominant with 22.5%. *Lactobacillus* and *Citrobacter* spp. were 12.5%; *Pseudomonas aeruginosa*, coagulase-negative Staphylococci, and *Serratia marcescens* were 10%; *Escherichia coli* was 7.5%; *Klebsiella* spp. and *Providencia* spp. were at 5%. The lowest prevalence was 2.5% of *Enterobacter* spp. and *Salmonella* spp. The threat of dissemination of isolated microorganisms is valid, since all devices evaluated in this study showed bacterial contamination of species associated to hospital-acquired infections. Special care should be taken when using electronic devices in healthcare settings in addition to disinfection to reduce the risk of transmission of bacterial agents. Further studies should evaluate the antibiotic susceptibility for better conclusive results since all isolated bacteria in this study were subjected to high resistance and were associated with nosocomial infections.

## 1. Introduction

Electronic devices have become one of the most essential accessories being used in hospitals and have transformed the medical environment. The use of electronic devices increases the communication and contact making healthcare delivery more efficient and effective [1]. Despite the better communication between healthcare professionals and patients, those devices represent a potential reservoir and source for transmission of infectious agents in clinical settings [2]. The healthcare-associated infections correspond to a major problem in hospitals, since they are related to a rate of morbidity and mortality of hospitalized patients thus constituting a negative charge both for patients and for public health [3–5]. Among the possible causes of hospital infections, the hands of healthcare providers, thermometers, and stethoscopes and other medical devices especially in sensitive departments of hospitals play an important part in transmission of pathogenic agents and constitute the source of multidrug-resistant organisms [6]. Due to the easy handling (portability, touchscreen nature) during healthcare provision and their use in all departments, including the units that involve patients with critical condition, it has been reported that electronic devices are considered fomites due to the potential conducive environment to the development of pathogens [7–9]. It is known that in a clinical environment, the hands constitute the source for the dissemination of the pathogens and play an important role in the transmission of hospital-acquired infections while providing healthcare without the disinfection and/or the proper hygiene [10, 11]. Studies have reported the contamination of stethoscopes, and other electronic devices were contaminated with various types of bacteria pathogens [8, 12, 13].

Although there is a wide range of literature reporting on hospital-acquired infections, few studies have been conducted to evaluate the possible contamination of electronic devices used by healthcare workers in Africa [14, 15]. To the best of our knowledge, no study in Rwanda has described the bacteriological status of electronic devices from which the implementation of electronic device surveillance systems could be developed. Therefore, the present work is aimed at describing and extending the understanding on electronic device contamination, the potential source of healthcare-associated infections, and providing reliable information about the spreading of pathogens through electronic devices especially in sensitive departments of Ruhengeri Referral Hospital.

## 2. Materials and Methods

### 2.1. Study Setting

It was a cross-sectional study conducted from November to December 2019 on electronic devices used by healthcare workers at Ruhengeri Referral Hospital within four different units: maternity, neonathology, intensive care, and theater room. Ruhengeri Referral Hospital provides quality healthcare to the population, training, clinical research, and technical support to district hospitals. It provides tertiary-level referral treatment and is known to be open 24 hours for emergency services.

### 2.2. Bacterial Isolation and Identification

Samples from different electronic devices were collected from the maternity unit (2 thermometers, 2 tensiometers, 1 computer, and 1 echography), intensive care unit (2 stethoscopes, 1 computer, 3 ventilators, 2 thermometers, and 2 tensiometers), neonathology unit (4 “newborn baby” incubators and 1 thermometer), and theater room (1 stethoscope, 1 surgical lighthead, 3 Datex Ohmeda, 2 variotherm, and 3 panda warmers). The samples were collected by means of moistened swabs in sterile saline solution by the technique of bearing on the surface of the devices and then placed into a transport medium (peptone water). To evaluate the effects of disinfection using 70% isopropyl alcohol, the second sample collection was carried out after decontamination of 5 swabbed devices (2 thermometers, 2 tensiometers, and 1 stethoscope) with 70% isopropyl alcohol. The swabbing was done after allowing the devices to dry for 10 minutes.

Samples were inoculated on the media (MacConkey and Blood Agar) appropriately with a wire loop using striking method, and the plates were aerobically incubated at 35-37°C for 18-24 hrs. To identify bacteria isolated in the present study, Gram staining and different tests were performed for biochemical test such as catalase test, coagulase test, sugar fermentation, indole production, urease test, motility test, and citrate test [16].

The authorization to conduct the research was obtained from Ruhengeri Referral Hospital and from INES-Ruhengeri.

## 3. Results

**Figure 1:**
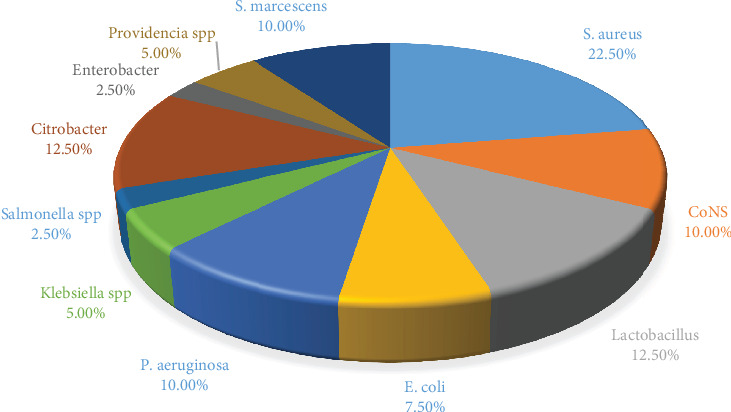
Bacterial frequency in collected samples from electronic devices.

*S. aureus* was the most predominant with 22.5%, followed by *Lactobacillus* and *Citrobacter* spp. at 12.5%. The lowest prevalence was 2.5% of *Enterobacter* spp. and *Salmonella* spp. (Figure 1).

**Figure 2:**
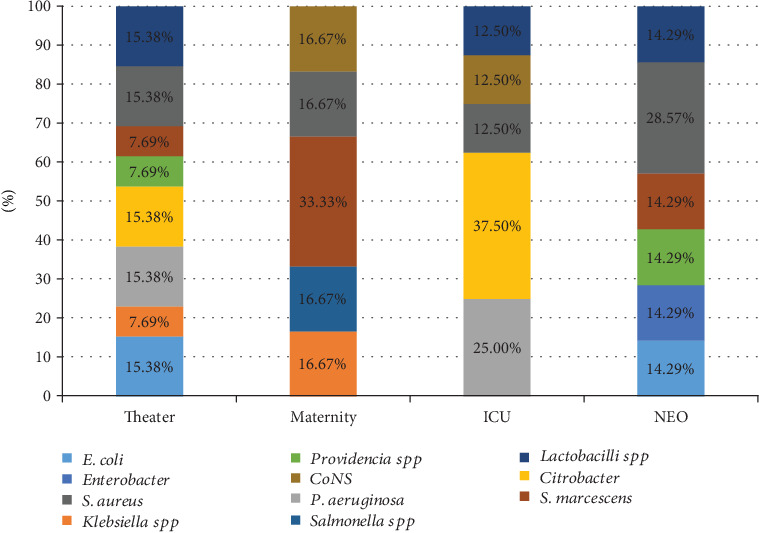
Types of bacteria isolated from electronic devices based on departments.

Figure 2 presents the prevalence of isolated bacteria from different units of Ruhengeri Referral Hospital. The theater room was the unit with the largest number of isolated bacteria, and the most predominant were *E. coli*, *P. aeruginosa*, *Citrobacter*, *S. aureus*, and *Lactobacilli* at the rate of 15.38%.

From the neonatology unit, *S. aureus* was the isolate with the highest frequency at 28.57%. In the maternity unit, isolated bacteria with the highest predominance were *S. marcescens* with the frequency of 33.33%. *Citrobacter* and *P. aeruginosa* had 37.50% and 25%, respectively, in the intensive care unit.

**Figure 3:**
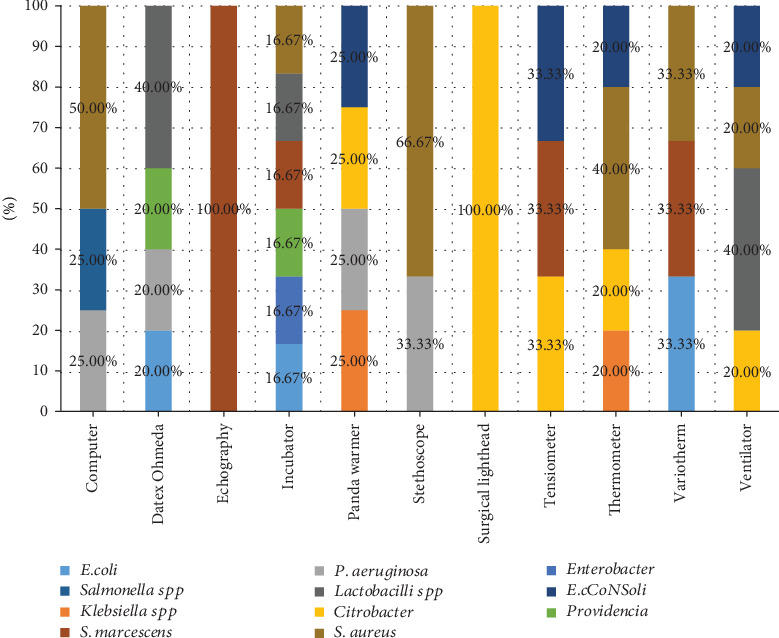
Percentage of isolated bacteria based on electronic devices.

Figure 3 describes the rate of contamination of different devices. There were 2 computers on which 3 types of bacteria were isolated including *S. aureus* at 50% and *Salmonella* spp. and *P. aeruginosa* at the rate of 25% each. Among all devices, 3 Datex Ohmeda were found to have the highest number of types of bacteria: *Lactobacilli* at the prevalence of 40%. *P. aeruginosa*, *E. coli*, and *Providencia* had 20% each. *S. marcescens* was isolated from 1 echography. From newborn baby incubators, 6 types of bacteria were identified including *E. coli*, *Enterobacter*, *Providencia*, *S. marcescens*, *Lactobacilli*, and *S. aureus* which had the same prevalence of 16.67%. Coagulase-negative *Staphylococcus* (*CoNS*), *Citrobacter*, *P. aeruginosa*, and *Klebsiella* spp. with the prevalence of 25% were isolated from 4 panda warmers. Two stethoscopes had 2 isolated bacteria: *S. aureus* and *P. aeruginosa* with the prevalence of 66.67% and 33.33%, respectively.

There was 1 surgical lighthead among the electronic devices from which *Citrobacter* was isolated. The findings also showed 3 types of bacteria isolated from 2 tensiometers including *CoNS*, *Citrobacter*, and *S. marcescens* with the prevalence of 33.33%. Three thermometers were found to have *S. aureus* with the rate of 40%; *CoNS*, *Citrobacter*, and *Klebsiella* spp. were identified with the prevalence of 20%. *E. coli*, *S. marcescens*, and *S. aureus* were isolated from 2 variotherm with the prevalence of 33.3%. *Lactobacilli* had the highest prevalence of 40% among bacteria isolated from 3 ventilators.

To test if the disinfectant alcohol 70% (*w*/*v*) can reduce microorganisms on electronic devices, 5 devices (2 thermometers, 2 tensiometers, and 1 stethoscope) were sampled before and after cleaning with alcohol. Before cleaning, all 5 devices showed bacterial growth with either single or mixed bacterial agents as described above. After cleaning, all five electronic devices were swabbed and samples were cultured and there was no bacterial growth observed.

## 4. Discussion

Understanding the causes of healthcare-associated infections constitutes one of the major components in preventive measures. In this pilot study, thirty electronic devices were used to analyze the potential source of contamination and possible transmission of microorganisms to highly sensitive patients. The greater portion (66.6%) of the samples was collected from the intensive care unit and theater room. The two units are among the health institution's units that need more care as the patients are more exposed to the great risk for acquiring nosocomial infections in view of their clinical condition and the variety of invasive procedures routinely performed [17, 18].

Samples collected from electronic devices in this study presented bacterial growth. All sampled devices were contaminated with either single or mixed bacterial agents. The rate of contamination of electronic medical devices analyzed was higher than the rate observed in similar studies [7, 13]. In this study, the bacterial contamination of electronic devices used by healthcare workers was similar to that reported by Bhat et al. [19], where from the 204 devices from medical and dental departments evaluated, 201 (98.53%) presented bacterial growth. Similar findings were reported by Selim and Abaza [20] where 40 mobiles phones from 4 different departments (laboratory, intensive care unit, dialysis unit, and triage area) of a healthcare setting were screened for the presence of bacterial contamination. All studied devices had one or more organisms. In comparison to the present study, Arora et al. [7] reported the lowest contamination, where from the 160 mobile devices of nursing staff evaluated, only 65 (40.62%) showed bacterial growth. The difference could be due to the fact that the current study analyzed different types of devices while they analyzed only mobiles devices.

In this study, the isolated microorganisms most commonly associated with healthcare acquired infections were *CoNS*, *S. aureus*, *Klebsiella* spp., *E. coli*, *P. aeruginosa*, *Enterobacter* spp., *Citrobacter* spp., *Lactobacillus* spp., *Providencia* spp. and *S. marcescens*. The most prominent identified microorganisms were *S. aureus*, *Citrobacter* spp. and *Lactobacillus* with 22.5%, 12.5%, and 12.5%, respectively. However, the study also found significant prevalence of *P. aeruginosa*, *CoNS*, *E. coli*, and *S. marcescens*. Compared to the study conducted by Worku et al. [21], the most isolated bacteria from 201 screened objects was *S. aureus* with 21.6% followed by CoNS and *E. coli* with 19.3% and 16%, respectively. In the study conducted in Jimma, Ethiopia, on 176 screened stethoscopes, they found that CoNS accounted for 103 (58.5%), *S. aureus* 79 (44.8%), and *Klebsiella* spp. 12 (6.8%) [22]. The difference in number and type of bacterial isolates between studies relies on variations in electronic devices analyzed, environmental sanitation, and hygiene practices of the clinical settings. The difference in the reported results was also observed in the study conducted in Nigeria where the isolated bacteria were Corynebacterium spp. (10%), Lactobacillus spp. (8%), Staphylococcus spp. (52%), and Streptococcus spp. (6%) from stethoscopes, sphygmomanometers, and clinical thermometers [15].

Humans constitute the largest reservoir of Staphylococci being present asymptomatically as normal human flora of the skin and mucous membrane. These microorganisms spread through direct contact with inanimate surfaces and cause infections in patients with critical clinical conditions [23, 24]. The greatest frequency in this study was expected and is explained by the fact that routinely the electronic devices are in contact with the skin, which is one of the habitats of species of the genus. This direct contact is possibly favoring the transfer of these microorganisms to medical devices [25]. *S. aureus* was reported by Humphreys et al. [26] as the main cause of infections of the bloodstream and one of the main causes associated with surgical site infections.

Although the antibiotic susceptibility test was not done in this study, the isolated bacteria are considered as healthcare-associated infections with a well-established high antibiotic resistance, especially in Africa [27]. In a study conducted by Worku et al. [21] on isolates from stethoscope, thermometer, and inanimate surfaces, the most multidrug-resistant bacteria were *S. aureus*, *Klebsiella* spp., and CoNS with 79%, 53.8%, and 47%, respectively. It has been noticed that the infections caused by *Klebsiella* have become difficult to treat due to the presence of plasmids encoding beta-lactamase enzyme conferring to the microorganism resistance to multiple antibiotics [28]. In the present study, *S. marcescens* showed a frequency of 10%. This is a Gram-negative bacterium responsible for a wide range of healthcare-associated infections in critically ill patients admitted in settings such as intensive care units, and it is an independent multiple multidrug-resistant bacterium [29].

Alcohol 70% (*w*/*v*) is routinely used as a disinfectant in clinical settings to reduce the bacterial colonization in surfaces and medical devices [30]. This study assessed its effectiveness on medical devices used at Ruhengeri Referral Hospital by culturing samples swabbed on those medical devices after cleaning with ethyl alcohol 70% (*w*/*v*). As reported above, before cleaning, culture media showed a high growth in cultured samples taken at Ruhengeri Referral Hospital. Interestingly, the same samples were collected and cultured after cleaning with ethyl alcohol 70% (*w*/*v*) and the results revealed no growth at all. These findings are similar to the study conducted by Graziano et al. [31] who demonstrated the effectiveness of disinfection with alcohol 70% (*w*/*v*) of contaminated surfaces with *S. marcescens*. They demonstrated the disinfectant effectiveness of alcohol 70% (*w*/*v*) applied directly to contaminated surfaces, presenting results which were equivalent when compared to the classically recommended method of decontamination, which consists of cleaning the surface prior to applying alcohol 70% (*w*/*v*). The same results were found by Bambace et al. [32], where while investigating on *Klebsiella pneumoniae*, *P. aeruginosa*, and *Streptococcus mutans*, they reported no recovery of studied strains in any of the surfaces cleaned by alcohol.

## 5. Conclusion

The threat of dissemination of isolated microorganisms is valid, since all devices evaluated in this study showed bacterial contamination of species associated to hospital-acquired infections. Restricting the use of the appliance is not feasible, since the benefits of those electronic devices are to provide quality healthcare services in all the departments of clinical setting. However, special care should be given to the very sensitive departments of the health settings to avoid hospital-acquired infections. Health professionals should be aware that devices of the hospital can contain and convey harmful microorganisms, which can be disseminated both in and outside the hospital environment. The use of electronic devices without cleaning with disinfectant ethyl alcohol 70% (*w*/*v*) increases the dissemination of microorganisms all over the clinical settings. Further studies should evaluate the antibiotic susceptibility for better conclusive results since all isolated bacteria in this study were subjected to high resistance and were associated with nosocomial infections.

## Data Availability

The raw data used to support the findings of this study are available from the corresponding author upon request.
